# Signs of Central Hypersensitivity, Stress, and Anxiety following Treatment for Breast Cancer: A Case Control Study

**DOI:** 10.1155/2021/5691584

**Published:** 2021-10-18

**Authors:** Ivana Leao Ribeiro, Ximena Gálvez González, Diego Lara Torres, Luz Alejandra Lorca, Snehil Dixit, Nicolás Yáñez Benavides, Francisco Ortega Gonzalez

**Affiliations:** ^1^Department of Kinesiology, Faculty of Healthy Sciences, Universidad Católica del Maule, Talca, Chile; ^2^Hospital del Salvador, Servicio de Salud Metropolitano Oriente, Santiago de Chile, Chile; ^3^Department of Medical Rehabilitation Sciences, College of Applied Medical Sciences, King Khalid University, Abha, Saudi Arabia; ^4^Subdepartamento de Oncología-Hospital Clínico Regional Valdivia, Unidad de Oncología-Clinica Alemana Valdivia, Chile; ^5^Facultad de Medicina, Universidad Católica del Maule, Talca, Chile; ^6^Servicio de Oncología Médica, Hospital Regional de Talca, Talca, Chile

## Abstract

**Background:**

With treatment for breast cancer, women treated may present significant sensory abnormalities in the upper extremity. However, there are no conclusive studies that have evaluated pressure pain thresholds (PPT) in the shoulder of postoperated women for breast cancer. The aim of this study was to compare PPT in the shoulder, stress, anxiety, depression symptoms, and quality of sleep among postoperated women for breast cancer (PO group) and asymptomatic women of shoulder pain (control group).

**Methods:**

40 women participated (*n* = 20, PO group, age: average ± standard deviation, 49.2 ± 8.3 years; body mass index (BMI): 27.5 ± 3.0 kg/cm^2^; surgery time: 22.2 ± 34.4 months; *n* = 20, control group, 46.9 ± 8.1 years; BMI: 26.8 ± 3.5 kg/cm^2^). The PPT was evaluated with a digital algometer at 32 points in the shoulder region and one control point in the tibialis anterior. Stress, anxiety, and depression were evaluated with the Depression, Anxiety and Stress Scale 21 (DASS-21) and the quality of sleep by the Pittsburgh Sleep Quality Index.

**Results:**

Significant differences were observed over 1.5 kgf/cm^2^ in 33 points evaluated (*p* < 0.01) with a small to high effect size (Cliff's delta range = 0.16; 0.92) and higher levels of anxiety and stress in the PO group (anxiety: median [first; third quartile], 5[3; 12.5]; stress: 9.7 ± 4.7 (7.8; 11.8)) in comparison with the control group (anxiety: 2.5[1; 4.8]; stress: 6.7 ± 3.31 (5.2; 8.3), (*p* < 0.05)). No significant differences were found between the groups in depression and sleep quality (*p* > 0.05).

**Conclusion:**

Postoperated women for breast cancer present hyperalgesia in the shoulder anterior and posterior region, low PPT in the tibialis anterior, and higher levels of stress and anxiety compared to the control group.

## 1. Introduction

Breast cancer is the most common cancer among women. A prevalence of 30% is estimated worldwide until the year 2023. Regarding the incidences in Latin America and the Caribbean, breast cancer accounts for 27% of new cases and 16% of cancer mortality, this being the second cause of death among women [1].

The treatments for breast cancer can generate important secondary complications mainly related to the chronic state of pain [2]. The types of pain are represented according to acute duration [3], after the end of the treatment or chronic, persistently evidenced in the area of surgery and reported up to 3 years after the end of the treatment [4]. Neuropathic pain produced mainly by peripheral nerve pathway injury is also frequently reported and is associated with anxiety and pain in the arm [5]. It is characterized by paresthesia, numbness, and allodynia [5], symptoms that can cause functional impairment, significant limitations in daily living activities, and compromised quality of life [6].

Topographic maps with various pressure pain threshold records have become useful tools to assess the stake of sensitization in various musculoskeletal conditions [7]. In the shoulder, a pressure pain map has been developed for people with rotator cuff disease [8] and also in the neck and shoulder region in breast cancer survivors, reporting bilateral hyperalgesia and central sensitization signs [9]. However, the latter focuses on limited areas considering fewer points and did not exclude signs of rotator cuff disease in the study's participants [9]. In this sense, it is interesting to carry out a map designed for people with rotator cuff disease in breast cancer survivors, since the literature reports that rotator cuff disease is associated in the long term, with postoperative breast cancer [10].

Likewise, symptoms and health conditions such as stress, anxiety, depression, and poor sleep quality are also reported by several studies in the literature after breast cancer treatment [11–13]. The presence of anxiety and depression has also been reported prior to breast surgery [12, 14], and symptoms are usually maintained at all stages of treatment [12]; however, there is not enough evidence regarding the presence of these symptoms in the long-term postoperative period. Regarding sleep disorders, these can seriously affect physical and mental well-being, and quality of life. Sleep disorders can be present and be even more serious in patients with diseases such as cancer [15, 16]. Difficulty sleeping adequately at night is one of the most prevalent symptoms during chemotherapy for breast treatment [17]. Likewise, the studies for not report the behavior of these variables in the following surgical treatment of breast cancer. Thus, the objective of the present study was to compare pressure pain thresholds in the shoulder, symptoms of stress, anxiety, depression, and quality of sleep between postoperated women of breast cancer and the control group of healthy women without painful shoulder symptoms.

## 2. Material and Methods

It corresponds to a case-control study, according to the Strengthening the Reporting of Observational Studies in Epidemiology (STROBE) [18].

### 2.1. Participants

Forty women with similar anthropometric characteristics participated in this study. The postoperative breast cancer group were recruited from the XXXX. The following inclusion criteria were considered: age between 18 and 70 years old, body mass index, BMI, ≤29.9 kg/m^2^, first diagnosis of cancer, and any type of surgical treatment [19] with a time of no less than one month after surgery. The exclusion criteria were previous musculoskeletal injuries in the shoulder, associated with subacromial pain syndrome as Jobe test, Neer test, Hawkins test, reported in a previous study [8], and pain range and external rotation test [20]. Women with the presence of metastases and upper limb lymphedema were also excluded. To determine the presence of lymphedema, a difference between both extremities over 2 cm was considered, evaluated using a tape measure [21, 22].

Twenty women from the control group were included in the study, considering the following criteria: between age 18 and 68 years old, asymptomatic regarding shoulder pain, with similar anthropometric characteristics (weight and height) to the postoperated breast group. Women who presented with previous musculoskeletal injuries in the shoulder to be evaluated, compatible with the subacromial impact symptoms mentioned above [20], and body mass index ≤ 29.9 kg/m^2^ were excluded.

The women that voluntarily agreed to participate in this study signed informed consent. This study was approved by the Scientific Ethics Committee of the Catholic University of Maule (number 16/2019 – 154/2019) and performed according to Resolution 466/12 of the National Health Council.

### 2.2. Sample Size

Sample size was based on the detection of at least 20% clinically significant differences in pressure pain threshold levels between both groups [23], with an alpha level of 0.05, a desired power of 80%, and an estimated interindividual coefficient with a variation of pain thresholds at pressure of 20% [23], considering 16 participants per group. To increase the power of study and objectifying sample losses, 20 participants were recruited and evaluated per group.

### 2.3. Procedures

The evaluations were performed in the Laboratory of Clinical Investigation in Kinesiology of Catholic University of Maule. For the evaluation of the pressure pain threshold, the randomization of 33 points to evaluate in each patient was considered through a webpage (http://www.randomization.com/) for both groups.

### 2.4. Evaluation of Pressure Pain Threshold

**Figure 1:**
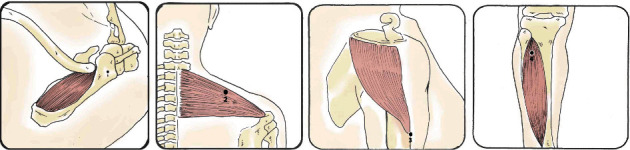
Distribution of fixed points (1-4).

**Figure 2:**
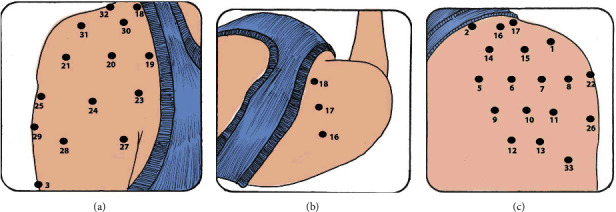
Distribution of the points in the anterior (a), superior (b), and posterior (c) regions of the shoulder (5-33).

A Wagner brand digital algometer was used to assess pressure pain threshold. The measurement was performed according to the topographic map proposed for patients with subacromial pain syndrome, where 32 points belong to the shoulder region and a control point in the tibialis anterior region. The procedure is described in full detail elsewhere [8], and Figures 1 and 2 represent fixed points and distribution around the shoulder area. Three measurements were performed for each point with 20 seconds of rest between each measurement to avoid temporal summation [7]. Each patient evaluated made a warning signal (hand raise) at the moment of feeling pain and thus stop the measurement to collect the value delivered by the algometer. The tip of the instrument was positioned perpendicular to the area being evaluated, and the pressure was maintained, which was progressively increased to 1 kg/seg. The same day interrater reliability measured by intraclass correlation coefficient of the pressure pain thresholds presents values from 0.76 to 0.98 in people and without shoulder pain, being considered moderate to excellent [24].

### 2.5. Evaluation of Pain Reported

The visual analog scale (VAS) was used to assess pain on the affected arm at rest [25], with a score that varies from 0 to 100 mm (0 = no pain). The VAS is a valid and reliable tool to assess pain in subjects with shoulder pain [26] and presents a minimum detectable difference of 1.3 points on the pain scale (95% confidence interval: 1.0-1.5) [27].

### 2.6. Evaluation of Stress, Anxiety, and Depression

To evaluate the variables of stress, anxiety, and depression, the DASS-21 scale was used, which reflects the feelings of the previous week. This scale has 21 items divided into three subscales: 7 questions related to stress, 7 questions related to anxiety, and 7 related to depression. In addition, there are 4 alternative responses, which range from 0 (“It does not describe anything that has happened to me or I felt during the week”) to 3 (“Yes, this happened to me a lot or almost always”). This scale has the advantage of being a self-reporting instrument, it is brief and easy to administer and answer, and its interpretation is simple [28]. In this study, the version in Spanish (for Chile) was used, which was linguistically validated, culturally adapted, translated, and adapted to Chilean Spanish [29].

### 2.7. Evaluation of Sleep Quality

To evaluate sleep quality, the Pittsburgh Sleep Quality Index (PSQI) was used, which assesses sleep quality and disturbances during the previous month [30]. The questionnaire has 19 self-assessment questions and 5 questions directed to the roommate or bedmate, being only the first of 19 questions used to obtain the global score. These questions are organized into seven components, such as subjective sleep quality, latency, duration, efficiency, sleep disturbances, use of sleep medication, and daytime dysfunction. The sum of score from all these components gives a total of 0 to 21 points: a score of less than 5 are called “sleep without problems,” between 5 to 7 points as “deserves medical attention,” and between 8 and 14 as “deserves medical attention and treatment,” and when the score is higher than 15 points, it is “serious sleep problems.” Therefore, the higher the score, the worse the sleep quality [30]. This questionnaire has been validated in the Spanish language [31] and used in the breast cancer population during chemotherapy [32].

### 2.8. Data Analysis

The statistical analysis was performed using an SPSS statistical package (version 25.00). The Kolmogorov-Smirnov test was used to determine the normality of the data; the variables with normal distribution (age, weight, height, BMI, surgery type, stress, DASS-21, and PSQI) are expressed as mean ± standard deviation (lower limit; upper limit of the 95% confidence interval), in both variables with nonnormal distribution (depression and anxiety) by median (minimum; maximum) [first quartile; third quartile].

The statistical analysis was performed at a 95% confidence level. A *p* value of *p* < 0.05 was considered statistically significant for all tests. Student's *t*-test was performed to detect differences in demographic characteristics, stress, DASS-21, and PSQI between groups. The nonparametric Mann-Whitney *U* test, which considers two independent samples, for stress and anxiety and to detect the differences in the pressure pain threshold scores using the points (from 1 to 33), between groups was performed. Finally, the Cliff's Delta Calculator program was used to quantify the effect size of the pressure pain threshold variable (kgf/cm^2^) of each point between the PO group and the control group, as reported in a previous study [22]. A Cliff's delta of 0 means no effect or no difference between the groups, while values close to -1.0 or +1.0 indicate an absence of overlap in the measurements between two groups. When a significant *p* value is obtained, the effect size close to +1.0 or -1.0 is considered important [33].

## 3. Results

**Figure 3:**
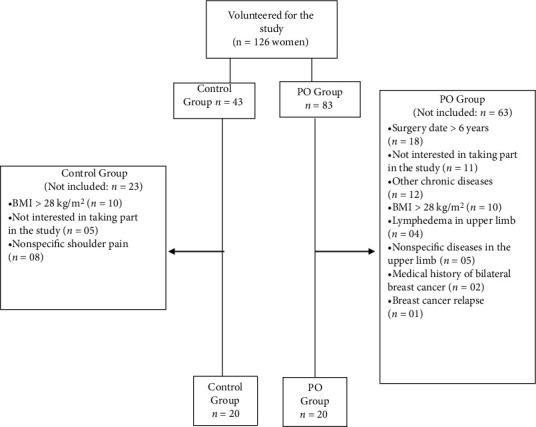
Flowchart of study participants.

Of the 40 participants in this study, 20 women (Figure 3) belong to the PO group, having an average age of 49.2 ± 8.3 years old, body mass index (BMI) 27.5 ± 3.0 kg/cm^2^, and an average of 22 months after breast cancer surgery.

**Figure 4:**
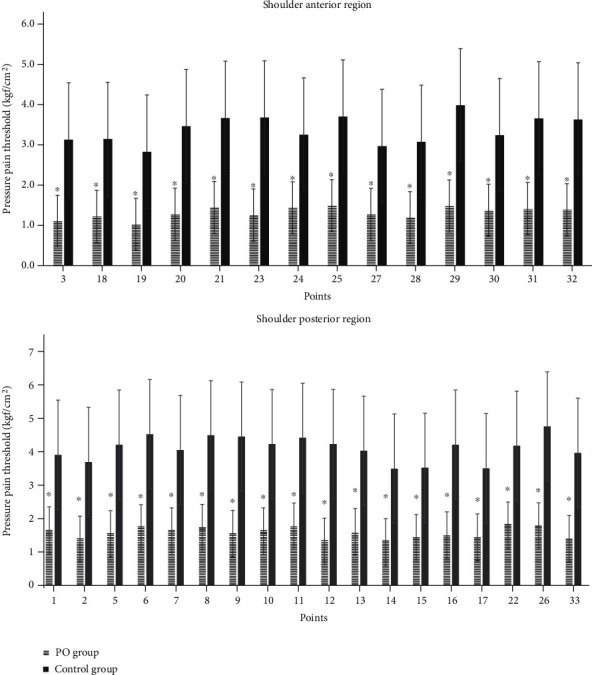
Distribution of assessed points at anterior and posterior shoulder region between groups. Legend: ^∗^*p* < 0.05 between groups; PO: postoperated breast cancer surgery group.

Regarding distribution of points in the shoulder anterior and posterior region, Figure 4 evidences low-pressure pain threshold at the PO group in all assessed points compared to the control group (Figure 4).

The control group was composed of 20 women, having an average age of 46.9 ± 8.1 years and BMI of 26.8 ± 3.5 kg/cm^2^. Of the postsurgery group, 19 participants (95%) were right dominant and one participant (5%) was left dominant. In the control group, 17 participants (85%) were right dominant and the remaining three (15%) were left dominant. Of participants of the PO group, eight (40%) had operation on the right side and 12 (60%) on the left side, 55% had conserving breast surgery, and 50% had axillary lymphadenectomy approach (Table 1).

In regard to the clinical characteristics of the study participants (Table 2), the PO group reported pain in the limb affected by the surgery during rest (range: 0-6) and showed higher levels of anxiety and stress compared to the control group (*p* < 0.05) with no differences for depression symptoms (*p* > 0.05). For the Pittsburgh Sleep Quality Index, no significant differences were found between the groups (*p* > 0.05).

Table 3 shows pressure pain thresholds in the shoulder region for both groups. Significant differences were observed over 1.5 kgf/cm^2^ between groups for all the points evaluated (*p* < 0.01). Among all points evaluated, the one that assesses central sensitivity (P4–tibialis anterior), the lowest sensitivity threshold was obtained in the PO group (PO: 3.4 kgf/cm^2^; control: 7.5 kgf/cm^2^).

## 4. Discussion

The main objective of the study was to compare pressure pain thresholds in the shoulder, symptoms of stress, anxiety, depression, and sleep quality between postoperated women for breast cancer and in asymptomatic women without a history of cancer. The results found allowed for characterizing the presence of low-pressure pain thresholds among all points evaluated in the shoulder when compared with the control group. In addition, it was possible to identify higher levels of stress and anxiety in the postoperative group up to approximately 2 years after breast cancer surgery. Regarding depression symptoms and sleep quality, no significant differences were reported between the two groups.

Low-pressure pain thresholds were reported in a previous study [9, 34], mainly in the shoulder and neck region up to 6 months after breast cancer surgery, specifically in the area of the trapezius, anterior deltoids, pectoralis major [9], and tibialis anterior [34]. Similar findings are evident in people with rotator cuff disease [8]. It is important to consider that this disease has low-pressure pain thresholds in the shoulder region and the presence of trigger points in the scapular muscles [35, 36].

Through the participants of the present study that did not present symptoms of rotator cuff disease when performing different tests prior to the evaluation, the low thresholds for pressure pain in the shoulder suggest the development of future symptoms of this disease of this population [10]. In the present study, a map previously used in people suffering from subacromial syndrome was considered [8]. Previous studies have described the general areas of sensitization in postoperative breast cancer patients around the pectoralis major, posterior deltoid, dorsal region [37], and over peripheral nerve trunks of the median, radial, and ulnar nerves [38]. However, the addition of a more accurate description of the distribution of pressure pain sensitivity is of interest to design specific rehabilitation programs. Regarding point distribution over the shoulder, it was evidenced that both anterior and posterior regions of postoperated women for breast cancer had lower pressure pain threshold in all assessed points suggesting a generalized pressure pain hyperalgesia in comparison to a control group. This is in line with a previous study that reported hypersensitivity distribution over the posterior region of the shoulder following breast cancer treatment [9]. With respect to anterior hypersensitivity observed, results suggest that participants may have future symptoms of subacromial pain syndrome [8, 10].

This study showed higher levels of stress and anxiety and no reported depressive symptoms in those breast cancer survivors up to approximately 2 years after the surgery was performed. However, a previous study [12] reported that participants diagnosed with breast cancer did not tend to suffer significant changes in the variables of stress, anxiety, depression, and sleep quality during different treatments of the disease or after them [12]. Other studies revealed negative emotional states in breast cancer survivors during the first year of treatment [39, 40]. Regarding depressive symptom results reported in the current study, we assumed that following breast cancer treatment, patients tend to develop positive adjustments as an adaptive behavior after treatment; those are in line with the period reported after surgery (mean of 22.2 months).

In relation to sleep quality, in this study, there were no significant differences between both groups, since the patients belonging to the control group reported sleeping a limited number of hours referring to the fact that they slept late and woke up early due to work reasons. Many of the patients in the control group also mentioned waking up during the night due to heat or cold and also to go to the bathroom. Previous literature shows that poor sleep quality is present in 85% of women with breast cancer; this is associated with the presence of low self-esteem, pain [41], and hopelessness [42].

The limitations of this study were mainly the surgery time, since it was varied; some patients had been postoperative 3 months, while others had 10 months. There is variability in the type of breast and axillary approach. Age and body mass index variability between patients were also considered a limitation of this study since that may have some negative influence in pain perception [43, 44]. These limitations could have a direct relationship with low-pressure pain thresholds and also with pain perception. However, in addition to not having differences in the demographic characteristics of the participants, only postoperated women for breast cancer presented lower pressure pain thresholds and higher levels of stress and anxiety. Other limitations related to comorbidities as fibromyalgia and other musculoskeletal diseases not associated to the shoulder were not considered and should be taken into account in future studies.

This study revealed important sensory alterations in the shoulder region and higher levels of stress and anxiety in the patients corresponding to the postoperative breast cancer group when compared with the control group of asymptomatic women of shoulder pain. These results would support the presence of central sensitization in those patients who showed low levels of pressure pain threshold.

Topographic maps of pain sensitivity to pressure provide spatial information on those most affected sites in patients, which could facilitate and direct eventual physical therapy interventions in more specific regions of the body. This information substrate can guide future studies investing sensory alterations in populations with other clinical conditions.

## 5. Conclusions

The study showed that postoperated women for breast cancer present hyperalgesia in the shoulder anterior and posterior region, low PPT in the tibialis anterior area, and higher levels of stress and anxiety compared to a control group. Current results suggest the need to increase the pain and emotional assessment following breast cancer treatment to design and further to assess the effectiveness of rehabilitation-specific programs.

## Figures and Tables

**Table 1:** Clinical characteristics of participants (*n* = 40).

	PO group (*n* = 20)	Control group (*n* = 20)
Dominance (R/L)	19/1	17/3
Affected side (R/L)	8/12	—
Time since surgery (months)	22.2 ± 34.4 (6.09; 38.31)	—
Type of breast surgery (CS/M)	11/9	—
Type of axillary approach (L/SLN)	10/9	—
Age (years)	49.2 ± 8.3 (45.3; 53.1)	46.9 ± 8.1 (43.1; 50.7)
Weight (kg)	68.0 ± 8.2 (64.1; 71.8)	67.1 ± 11.1 (61.9; 72.2)
Height (cm)	156.9 ± 4.6 (154.7; 159.1)	157.9 ± 5.8 (155.1; 160.6)
Body mass index (kg/m^2^)	27.5 ± 3.0 (26.1; 28.9)	26.8 ± 3.5 (25.2; 28.4)

PO: postoperated breast cancer surgery group; CG: control group; R: right; L: left; CS: conserving surgery; M: mastectomy; L: lymphadenectomy; SND: sentinel lymph node biopsy. Values are expressed as mean ± standard deviation (lower and upper limit of 95% confidence interval) and frequency, *p* > 0.05 when comparing groups by Student's *t*-test.

**Table 2:** Perceived pain at rest, symptoms of depression, anxiety, stress, and sleep disturbance among participants (*n* = 40).

	PO group (*n* = 20)	Control group (*n* = 20)
VAS (0-100 mm)	2.1 ± 2.8	—
Depression (0-21)	3 (0; 9)[1; 5.8]	2.5 (0; 2)[1; 5.5]
Anxiety (0-21)	5 (0; 2)[3; 12.5]^∗^	2.5 (0; 1)[1; 4.8]
Stress (0-21)	9.7 ± 4.7 (7.8; 11.8)^∗^	6.7 ± 3.31 (5.2; 8.3)
DASS-21 (0-63)	20.2 ± 11.5 (14.8; 25.5)	13.8 ± 9.3 (9.5; 18.1)
PSQI (0-21)	8.8 ± 4.0 (6.9; 10.7)	8.2 ± 3.2 (6.7; 9.7)

VAS: visual analog scale at rest; DASS-21: depression, anxiety, and stress scales; PSQI: Pittsburgh sleep quality index. Values are expressed as mean ± standard deviation (lower and upper limit of 95% confidence interval) and median (minimum; maximum) [first quartile-third quartile]. ^∗^*p* < 0.05 when comparing groups by Student's *t*-test.

**Table 3:** Pressure pain thresholds of each point in the postoperated breast cancer surgery group (*n* = 20) and control group (*n* = 20).

	PO group (*n* = 20)	Control group (*n* = 20)	*U* test	*p* value	Cliff's delta
Point 1	1.4 (0.6; 4.0)[1.0; 2.2]^∗^	4.0 (1.4; 6.4)[3.0; 4.8]	37.5	<0.01	-0.78
Point 2	1.5 (0.7; 21)[1.0; 1.7]^∗^	3.7 (1.4; 6.4)[2.4; 4.8]	13.0	<0.01	-0.91
Point 3	0.9 (0.6; 2.5)[0.7; 1.3]^∗^	2.9 (0.8; 6.0)[2.4; 4.0]	27.5	<0.01	-0.78
Point 4	3.4 (0.9; 9.4)[2.0; 5.2]^∗^	7.5 (3.7; 12.4)[6.3; 10.2]	37.5	<0.01	0.16
Point 5	1.5 (0.6; 3.9)[0.9; 1.9]^∗^	4.0 (1.6; 9.7)[2.9; 5.9]	22.5	<0.01	-0.84
Point 6	1.6 (0.6; 3.9)[1.2; 2.2]^∗^	4.7 (1.2; 8.9)[3.0; 6.0]	29.5	<0.01	-0.85
Point 7	1.6 (0.8; 3.2)[1.2; 2.0]^∗^	4.3 (1.8; 6.4)[3.0; 5.2]	23.5	<0.01	-0.85
Point 8	1.6 (0.5; 3.8)[1.4; 2.1]^∗^	4.6 (2.1; 7.1)[3.2; 6.3]	20.0	<0.01	-0.91
Point 9	1.4 (6.6; 4.0)[1.0; 2.1]^∗^	4.3 (1.4; 6.4)[3.0; 5.0]	24.0	<0.01	-0.86
Point 10	1.5 (0.3; 4.4)[1.0; 2.3]^∗^	4.2 (1.1; 9.4)[2.5; 5.6]	37.0	<0.01	-0.82
Point 11	1.6 (0.7; 3.9)[1.2; 2.2]^∗^	4.7 (1.6; 7.4)[3.0; 5.4]	28.5	<0.01	-0.84
Point 12	1.2 (0.4; 2.6)[0.9; 1.6]^∗^	3.9 (1.5; 9.5)[2.6; 5.2]	13.5	<0.01	-0.92
Point 13	1.4 (0.6; 3.7)[1.0; 2.3]^∗^	4.2 (1.2; 7.0)[2.4; 5.3]	37.0	<0.01	-0.87
Point 14	1.3 (0.6; 3.0)[1.0; 1.5]^∗^	3.3 (1.6; 6.4)[2.6; 4.5]	9.0	<0.01	-0.88
Point 15	1.3 (0.6; 3.3)[1.0; 1.6]^∗^	3.6 (1.5; 5.5)[2.6; 4.4]	22.0	<0.01	-0.82
Point 16	1.4 (1.1; 2.3)[1.1; 1.8]^∗^	4.1 (1.4; 7.3)[2.5; 6.0]	20.5	<0.01	-0.85
Point 17	1.5 (0.6; 2.5)[1.2; 1.7]^∗^	3.5 (1.7; 5.8)[2.6; 4.5]	9.5	<0.01	-0.86
Point 18	1.1 (0.6; 2.4)[0.9; 1.4]^∗^	2.9 (1.4; 6.4)[1.9; 4.0]	22.5	<0.01	-0.77
Point 19	1.0 (0.7; 1.7 [0.7; 1.2]^∗^	2.6 (1.1; 5.2)[2.2; 3.4]	9.5	<0.01	-0.89
Point 20	1.2 (0.6; 2.9)[0.8; 1.3]^∗^	2.9 (1.5; 7.6)[2.3; 4.1]	18.0	<0.01	-0.82
Point 21	1.4 (0.6; 3.8)[0.9; 1.6]^∗^	3.7 (1.5; 5.9)[2.9; 4.4]	21.5	<0.01	-0.83
Point 22	1.6 (0.6; 3.9)[1.3; 2.4]^∗^	4.5 (1.8; 6.8)[2.9; 5.4]	31.5	<0.01	-0.82
Point 23	1.1 (0.7; 2.7)[0.9; 1.5]^∗^	3.4 (1.7; 6.8)[2.6; 4.3]	14.0	<0.01	-0.90
Point 24	1.2 (0.6; 4.1)[1.0; 1.7]^∗^	3.1 (1.2; 5.6)[2.6; 4.0]	28.5	<0.01	-0.72
Point 25	1.3 (0.6; 4.2)[0.9; 1.8]^∗^	3.5 (1.2; 5.9)[3.0; 5.2]	37.0	<0.01	-0.81
Point 26	1.5 (0.8; 3.6)[1.2; 2.6]^∗^	4.5 (1.8; 9.3)[3.6; 6.2]	27.0	<0.01	-0.83
Point 27	1.0 (0.5; 3.7)[0.7; 1.5]^∗^	2.7 (1.2; 6.8)[2.1; 3.5]	44.5	<0.01	-0.73
Point 28	1.0 (0.6; 2.9)[0.7; 1.5]^∗^	2.9 (1.4; 5.6)[1.9; 3.7]	27.5	<0.01	-0.81
Point 29	1.4 (0.8; 4.2)[0.9; 1.8]^∗^	4.0 (1.1; 6.5)[2.9; 5.5]	36.5	<0.01	-0.85
Point 30	1.2 (0.8; 3.3)[0.8; 1.7]^∗^	3.1 (1.0; 6.5)[2.4; 4.3]	41.5	<0.01	-0.77
Point 31	1.2 (0.6; 3.1)[0.9; 1.9]^∗^	3.4 (1.4; 6.6)[2.8; 4.3]	22.0	<0.01	-0.84
Point 32	1.2 (0.6; 2.6)[0.9; 1.8]^∗^	3.1 (1.6; 7.2)[2.7; 4.5]	13.0	<0.01	-0.90
Point 33	1.3 (0.6; 3.1)[0.9; 1.7]^∗^	3.7 (1.4; 8.2)[2.8; 4.4]	16.5	<0.01	-0.88

Values are expressed as median (minimum; maximum) [first quartile-third quartile]. ^∗^Significant differences between groups (*p* < 0.01), Mann-Whitney test.

## Data Availability

The data used to support the findings of this study are available from the corresponding author upon request.
